# Neuroendocrine carcinoma of the gallbladder concomitant with adenocarcinoma of the sigmoid colon: A rare case report

**DOI:** 10.1016/j.amsu.2021.102359

**Published:** 2021-05-06

**Authors:** Mohamed Chablou, Yassine Mabrouk, Khalil Maamar, Rachid Jabi, Mohammed Bouziane

**Affiliations:** General Surgery Department, Mohammed VI University Hospital/Faculty of Medicine and Pharmacy, Mohammed First University, Oujda, Morocco

**Keywords:** Neuroendocrine, Carcinoma, Gallbladder, Concomitant, Case report

## Abstract

**Introduction and importance:**

Neuroendocrine carcinoma of the gallbladder is rare and aggressive, its diagnosis is based on pathologic and immunohistochemical examination, currently there is no standard treatment for this tumor. Its concomitant occurrence with adenocarcinoma of the sigmoid colon is exceptional.

**Case presentation:**

This case report describes a rare case of neuroendocrine carcinoma of the gallbladder that occurred concomitantly with sigmoid colon adenocarcinoma. The diagnosis of neuroendocrine carcinoma was established postoperatively by pathological and immunohistochemical examination. The biopsy of the colonic process confirmed the preoperative diagnosis. A laparotomy found a vesicular fundus process, peri-hilar adenopathy, metastasis of the liver segment V, and a sigmoid colon process. The patient underwent a cholecystectomy extended to segments IVb and V, with lymph node dissection and sigmoid colectomy. The postoperative follow-up was uneventful, and the length of hospitalization was seven days. The patient is currently undergoing adjuvant chemotherapy.

**Discussion:**

Neuroendocrine carcinoma of the gallbladder is extremely rare, with few case reports, its occurrence concomitantly with adenocarcinoma of the sigmoid colon is exceptional. Histology and immunohistochemistry confirm the diagnosis, surgery is essential in the management of patients affected by this tumor. The presence of synchronous metastases causes doubt about their primitive, hence the primordial place of surgery and histological examination to adopt adequate management of the patients.

**Conclusion:**

We underline the rarity of this neuroendocrine tumor and the importance of histology and surgery in its management.

## Highlights

• Neuroendocrine carcinoma of the gallbladder is a rare and aggressive tumor usually diagnosed at an advanced stage.
• The patient presented with a vague abdominal pain and bleeding per rectum.
• The final diagnosis is made upon histological and immunohistochemical examination.
• The concomitant occurrence neuroendocrine neoplasm of the gallbladder and adenocarcinoma of the sigmoid is exceptional.

## 1 Introduction

Gallbladder masses are commonly encountered on diagnostic imaging examinations. Distinguishing between benign and malignant conditions is critical, benign masses include Gallbladder Polyps, adenomyomatosis, granular cell tumor, and neurofibroma [1]. Approximately 98% of gallbladder malignancies represent primary carcinoma [1]. Neuroendocrine neoplasms (NEN) are recognized as a group of rare and heterogeneous tumors that originate from neuroendocrine cells found throughout the body [2]. Mainly in the gastrointestinal tract, lungs, and thyroid [3]. The neuroendocrine gallbladder neoplasm (GB-NEN) is extremely rare in clinical practice with few case reports. Neuroendocrine carcinoma of the gallbladder (GB-NEC) is defined as a poorly differentiated NEN and includes small cell carcinoma and large cell carcinoma. GB-NEC is an aggressive malignant tumor with a poor prognosis, usually diagnosed at an advanced stage resulting in early liver invasion and lymphatic metastasis. Early detection and diagnosis, as well as well-conducted treatment, are necessary to improve this prognosis, which requires more effort by research teams.

Colorectal cancer (CRC) is the third most common cancer worldwide [4]. Approximately 20% of patients with CRC already have metastases at diagnosis, and this figure has been stable over the last two decades [5].

The concomitant occurrence of neuroendocrine carcinoma of the gallbladder and adenocarcinoma of the sigmoid colon is very little reported.

This work has been reported in line with the SCARE 2018 criteria [6].

### 1.1 Case presentation

**Fig. 1:**
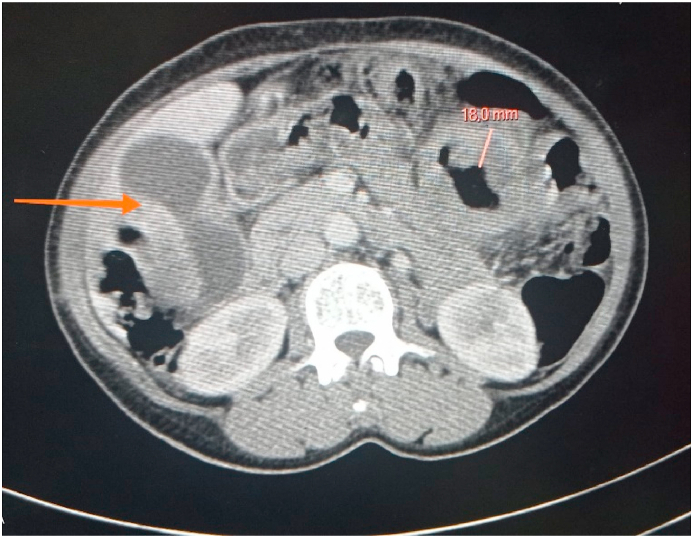
Abdominal CT scan image showing gallbladder and sigmoid colon process.

A 59-year-old woman was admitted to our hospital for pains in her right hypochondrium for four months, with rectal bleeding of average abundance for the last 15 days with a weight loss of 8 kg over four months. Abdominal examination revealed tenderness in the right hypochondrium, there was no palpable mass. The digital rectal examination found stool with blood. The rest of the physical examination was normal. Abdominal ultrasound showed a fusiform gallbladder with cholesterolic polyps. The thoracic and abdominopelvic computerized tomography (CT) scan found an irregular thickening, with infiltration of surrounding fat and mesenteric lymphadenopathy, and suspicious parietal thickening of the gallbladder. No metastasis was revealed. (Fig. 1).

**Fig. 2:**
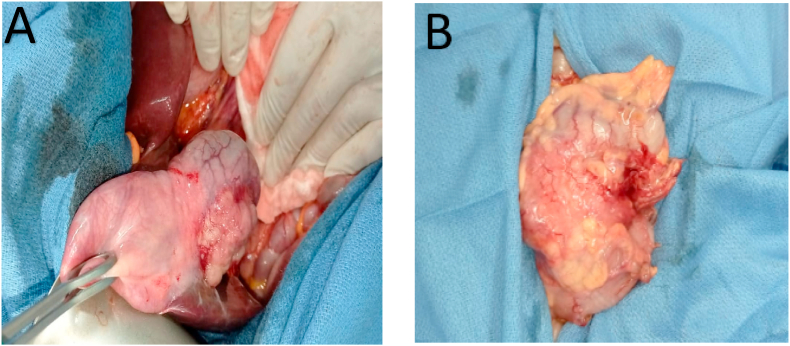
A) Intraoperative image of the gallbladder mass; B) Intraoperative image of the sigmoid colon tumor.

Eso-gastro-duodenal fibroscopy was normal and the colonoscopy found a circumferential sigmoid mass at 25–35 cm from the anal verge. The pathologic result of the biopsy of the sigmoid mass showed a well-differentiated infiltrating adenocarcinoma. Faced with a suspicious gallbladder, with a sigmoid colon adenocarcinoma with and the absence of metastasis, surgery was decided and a laparotomy was performed, the intraoperative exploration found a mass of the gallbladder fundus with multiple peri-hilar lymphadenopathies with an infracentimetric metastasis of the segment V of the liver, as well as a stenosing sigmoid tumor (Fig. 2).

**Fig. 3:**
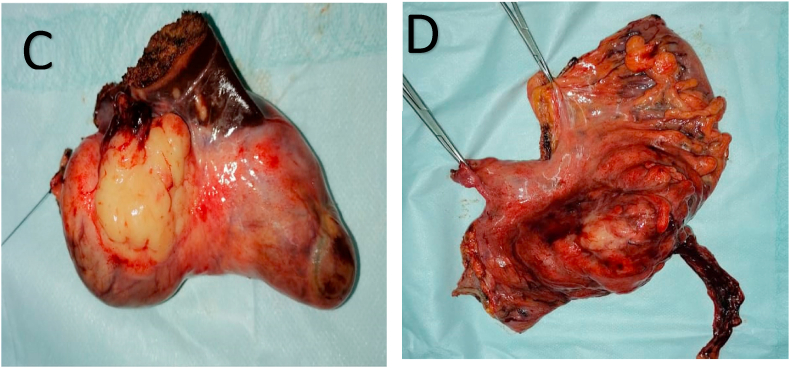
C) Image showing the resected gallbladder; D) Image showing the resected sigmoid colon.

A radical cholecystectomy with lymphadenectomy, and a sigmoid colectomy with a manual end-to-end colorectal anastomosis (Fig. 3).

The postoperative follow-up was without incident and the patient was discharged from the hospital on day 7. The pathologic results were as follows:

For the gallbladder: a large cell high-grade carcinoma neuroendocrine carcinoma (G3) invading the liver with a positive expression of chromogranin A, Synaptophysin, and CD56. Ki67 was around 80%; Lymph node metastases (8 N+/8 N).

For the sigmoid colon: a well-differentiated, infiltrating adenocarcinoma with lymph node metastases (2 N+/28 N). The tumor was classified as pT3N1b (AJCC 2019).

## 2 Discussion

Neuroendocrine Neoplasms (NENs) are rare, they predominate mainly in the gastrointestinal tract and respiratory tract, accounting for 66% and 31% of all NENs, respectively [7]. Their incidence is about 5.25/100,000 [8]. NENs in the gallbladder account for only 5% of these [9]. Their age of onset is between 38 and 81 years, and there is a significantly higher incidence among women [10]. The classification of neuroendocrine tumors is ambiguous, so experts from the International Agency for Research on Cancer (IARC) and the World Health Organization (WHO) have proposed a classification system for NENs in 2018 [11].

The approved classification for neuroendocrine tumors (NET) of the gastroenteropancreatic system (GEP) adopted by the WHO is commonly used by physicians [12] and is based on the rate of proliferation of the tumor, assessed using mitotic counts or the Ki-67 marker index. There are two main categories of NEN GEP: well-differentiated neuroendocrine tumors (NETs) include (G1) typical carcinoid, (G2) atypical or malignant carcinoid. (G3) Poorly differentiated neuroendocrine tumors include high-grade carcinoma, small-cell, and large-cell types [13].

Neuroendocrine carcinoma (NEC) of the gallbladder (GB) is rare and comprises only 0.2% of all GB tumors [14].

Its diagnosis is difficult preoperatively before histological evidence is available, as the clinical manifestations are not very specific, so the pain in the right hypochondrium and weight loss which are common, are also present in other gallbladder diseases such as adenocarcinoma.

Tumor markers that include carbohydrate antigen (CA) 19–9, carcinoembryonic antigen (CA) 19–9, carcinoembryonic antigen (CA) 19–9, carcinoembryonic antigen (CA) 19–9, and CA125 are often negative [15]. Which is the case of our patient. The imaging data are not specific, so the ultrasound may show a thickening, polyp, vesicular lithiasis, or ascites. CT and MRI scans cannot give a definite diagnosis but can show a thickening, a necrotic shadow in the most advanced lesions [16], locoregional adenopathy, or metastases without being able to predict their nature. Studies interpreting the results of CT and MRI scans in neuroendocrine gall bladder neoplasms have reported that the enhancement of GB-NEN is slightly more evident compared to that of adenocarcinoma [9]. Endoscopic retrograde cholangiography can reveal a process within the gallbladder and provide information about the condition of the main bile duct. Radiological examinations used at an early stage help to establish a staging and treatment strategy [17].

Other complementary examinations such as PET scans and bone scans are used to look for metastatic sites. In our patient, sub-centimetric mesenteric adenopathy was found, although it is unclear whether they were of colonic or vesicular origin. At present, the diagnostic certainty of neuroendocrine carcinoma of the gallbladder is based on the result of anatomopathological examination and immunohistochemical staining. Common biomarkers of immunohistochemistry are chromogranin A and synaptophysin, with positive rates of 91.9% and 84.8% [18]. Also, histological findings allow the classification and staging of the tumor.

Neuroendocrine carcinoma of the gallbladder is an aggressive tumor generally diagnosed at an advanced stage, and the therapeutic strategy depends on the stage of the tumor. There is currently no consensus on the management of patients with NEC GB, but surgery remains the primary treatment for this tumor, to have an R0 resection and improve the patient's quality of life [19]; thus at the early stage in situ, a cholecystectomy alone is sufficient. [20], Advanced tumors require large resections, with lymph node removal, see resection of liver metastases if possible [19], Chemotherapy is a significant alternative treatment for patients who cannot undergo surgery [21]. Although neuroendocrine carcinoma of the gallbladder is highly invasive and causes early lymph node metastases, surgery followed by concomitant radiochemotherapy is effective in prolonging survival [22].

The prognosis of gallbladder NEC is very poor, our patient had a grade 3 neuroendocrine carcinoma, with liver and lymph node metastases, as well as a high Ki67 index (80%), these are factors of poor prognosis [23].

Adenocarcinoma of the sigmoid colon is common, and its management is well known by doctors, its occurrence concomitantly with neuroendocrine carcinoma of the gallbladder, which is rare, is very exceptional. The presence of synchronous metastases can cause doubt as to their primitive; colic or vesicular, hence the primordial place of surgery and histological examination to adopt adequate management of the patients. Our patient had a neuroendocrine liver metastasis of segment V, and the lymph node dissection of group 8A and the hepatic pedicle found neuroendocrine carcinoma metastases, and the lymph node dissection of the sigmoid colectomy found metastatic lymph nodes of colonic adenocarcinomatous origin. The patient received her first course of adjuvant chemotherapy 15 days before the submission of this work.

## 3 Conclusion

Neuroendocrine carcinoma of the gallbladder is a very rare aggressive tumor, usually diagnosed at an advanced stage. Therefore, early detection, correct diagnosis, and reasonable treatment of these tumors will help to prolong the quality of life of affected patients. The clinical manifestations are atypical and the majority of laboratory and imaging examinations do not provide any specificity. The diagnosis of neuroendocrine carcinoma of the gallbladder depends on pathologic and immunohistochemical examinations, and surgery plays a key role in the management of these tumors. At present there is no consensus concerning the management of affected patients, hence the need for more research and exchange of the different treatment methods used by practitioners to improve the prognosis of these tumors.

Histology remains of prime importance in determining the origin of synchronous metastases in cases of concomitant neuroendocrine carcinoma of the gallbladder and another cancer different from another organ.

## Patient perspective

The procedure of surgery was explained to the patient with all advantages and possible complications. He agreed on the procedure and informed consent was taken from her.

## Ethics approval

No ethical approval necessary.

## Source of funding

The author(s) received no financial support for the research, authorship and/or publication of this article.

## Author contribution

Mohamed Chablou; Writing, review and editing of the manuscript.

Yassine Mabrouk, Khalil Maamar ;contributed for diagnose and treatment of the patient.

Rachid Jabi, Mohamed Bouziane: Review, Supervision and surgeons of the patient.

Registration of research studies: Our paper is a case report; no registration was done for it.

## Research registration number

1. Name of the registry:
2. Unique Identifying number or registration ID:
3. Hyperlink to your specific registration (must be publicly accessible and will be checked):

## Guarantor

Guarantor: Mohamed Chablou.

## Consent of the patient

Written informed consent was obtained from the patient for publication of this case report and accompanying images. A copy of the written consent is available for review by the Editor-in-Chief of this journal on request.

## Provenance and peer review

Not commissioned, externally peer reviewed.

## Declaration of competing interest

The authors declared no potential conflicts of interests with respect to research, authorship and/or publication of the article.

## References

[1] McKnight T., Patel A. (2012). Gallbladder masses: multimodality approach to differential diagnosis. J Am Osteopath Coll Radiol.

[2] Kunz P.L. (2015). Carcinoid and neuroendocrine tumours: building on success. J. Clin. Oncol..

[3] Hauso O., Gustafsson B.I., Kidd M., Waldum H.L., Drozdov I., Chan A.K.C., Modlin I.M. (2008). Neuroendocrine tumour epidemiology: contrasting Norway and North America. Cancer.

[4] Parkin D.M., Bray F., Ferlay J., Pisani P. (2005). Global cancer statistics, 2002. CA Cancer J Clin.

[5] Riihimäki M., Hemminki A., Sundquist J., Hemminki K. (2016). Patterns of metastasis in colon and rectal cancer. Sci. Rep..

[6] Agha Riaz A., Borrelli Mimi R., Farwana Reem, Koshy Kiron, Fowler Alexander J., Orgill Dennis P., For the SCARE Group (2018). The SCARE 2018 statement: updating consensus Surgical Case Report (SCARE) guidelines. Int. J. Surg..

[7] Gustafsson B.I., Kidd M., Modlin I.M. (2008). Neuroendocrine tumours of the diffuse neuroendocrine system. Curr. Opin. Oncol..

[8] Ayub F., Saif M.W. (2017). Neuroendocrine tumour of the cystic duct: a rare and incidental diagnosis. Cureus.

[9] Nishigami T., Yamada M., Nakasho K. (1996). Carcinoid tumour of the gall bladder. Intern. Med..

[10] Schwartz L.H., Black J., Fong Y. (2002). Gallbladder carcinoma: findings at MR imaging with MR cholangiopancreatography. J. Comput. Assist. Tomogr..

[11] Rindi G., Klimstra D.S., Abedi-Ardekani B. (2018). A common classification framework for neuroendocrine neoplasms: an International Agency for Research on Cancer (IARC) and World Health Organization (WHO) expert consensus proposal. Mod. Pathol..

[12] Klimstra D.S., Kloppell G., La Rosa S. (2019). Classification of neuroendocrine neoplasms of the digestive system.

[13] Luttges J. (2011). What's new? The 2010 WHO classifcation for tumours of the pancreas. Pathologe.

[14] Eltawil K.M., Gustafsson B.I., Kidd M., Modlin I.M. (2010). Neuroendocrine tumours of the gallbladder: an evaluation and reassessment of management strategy. J. Clin. Gastroenterol..

[15] Neyaz A., Husain N., Gupta S., Kumari S., Arora A., Awasthi N.P., Malhotra K.P., Misra S. (2018). Investigation of targetable predictive and prognostic markers in gallbladder carcinoma. J. Gastrointest. Oncol..

[16] Adachi T., Haraguchi M., Irie J., Yoshimoto T., Uehara R., Ito S., Tokai H., Noda K., Tada N., Hirabaru M. (2016). Gallbladder small cell carcinoma: a case report and literature review. Surg Case Rep.

[17] Liu W., Chen W., He X., Qu Q., Hong T., Li B. (2017). Cholecystectomy with gallbladder bed cautery might be sufficient for T1bN0M0 neuroendocrine carcinoma of gallbladders: cases report and literature review. Medicine (Baltim.).

[18] Soga J. (2003). Carcinoids and their variant endocrinomas. An analysis of 11842 reported cases. J. Exp. Clin. Canc. Res..

[19] Liu W., Chen W., Chen J., Hong T., Li B., Qu Q., He X. (2019). Neuroendocrine carcinoma of gallbladder: a case series and literature review. Eur. J. Med. Res..

[20] Sun Y.W., Liu D.J. (2011). Neuroendocrine carcinoma of the gallbladder. Chin J Pract Surg.

[21] Iype S., Mirza T.A., Pmpper D.J., Bhattacharya S., Feakins R.M., Kocher H.M. (2009). Neuroendocrine tumours of the gaHbladder: three cases and a review of the literature. Postgrad. Med..

[22] Shoushtari A.N., Bluth M.J., Goldman D.A., Bitas C., Lefkowitz R.A., Postow M.A., Munhoz R.R., Buchar G., Hester R.H., Romero J.A. (2017). Clinical features and response to systemic therapy in a historical cohort of advanced or unresectable mucosal melanoma. Melanoma Res..

[23] Shapera E., Bitting C. (2019). Survival: a rare outcome in large cell neuroendocrine carcinoma of the gallbladder. Acta Gastroenterol Belg.

